# Implementation of nanographene oxide combined with mineral trioxide aggregate and hydroxyapatite biopolymer in regeneration of critical-sized bone defect in rats

**DOI:** 10.1038/s41598-025-17233-5

**Published:** 2025-09-12

**Authors:** Amr H. Abdallah, Nesrine Ebrahim, Samar Saeed, Ahmed N. Abdallah, Samar H. Elsharkawy, Ahmed I. Abdelgalil, Wahid S. El Ghoul, Yasser M. A. Mohamed, Ashraf A. Shamaa

**Affiliations:** 1 Department of Surgery, Anesthesiology and Radiology- Faculty of Veterinary Medicine, Cairo University, Giza, 12211 Egypt; 2 Faculty of Medicine, Benha University. Benha National University, Faculty of Medicine, Benha, Egypt; 3 Keele University, Stoke-on-Trent, UK; 4 National Institute of Laser Enhanced Sciences, Cairo University, Giza, 12613 Egypt; 5 Department of Hormones, Veterinary Research Institute, National Research Centre, 33 El-Bohouth St, P.O. Box 12622, Dokki, Giza, Egypt; 6 Photochemistry Department, National Research Centre, 33 El-Bohouth St, P.O. Box 12622, Dokki, Giza, Egypt

**Keywords:** Critical-sized bone defects, Bioactive composite, Nano-graphene, Hydroxyapatite, Mineral trioxide aggregate, Biotechnology, Cell biology, Chemical biology, Developmental biology, Structural biology, Medical research, Materials science, Nanoscience and technology

## Abstract

Critical-sized bone defects (CSBDs) are causing a significant challenge in orthopedic surgery for their inability to heal spontaneously, demanding innovative biomaterials to enhance bone formation. Current therapies, as autografts and allografts, are restricted by donor site morbidity and immune rejection. The current study presents a novel, biocompatible composite material formed of nano-graphene oxide (nGO), mineral trioxide aggregate (MTA), and hydroxyapatite (HAp) and designed to synergistically control the unique characters of each component. The novelty of this composite is due to its composition as it formed via the combination of nGO for enhancement of the mechanical strength and the cell proliferation, MTA for its higher bioactivity and its ability for cement formation, while the HAp having optimum biocompatibility and osteoconductivity, this synergistic interaction was not previously explored for CSBD repair. The current study utilized a rat model of critical-sized radial bone defects. The nGO/MTA/HAp composite was manufactured by consuming a modified Hummer’s method for nGO, combined with commercially available MTA and HAp. Radiographic and computed tomography (CT) evaluation at 2-, 4-, and 8-weeks post-operation elaborating the progressive bone formation in the treated group compared to minimal changes in the untreated group. Histopathological examination demonstrated strong composite integration, massive cellular infiltration, and strong signs of osteoblast differentiation, causing approximately 75–85% defect closure at the 8th week. The current study highlights the potential of the nGO/MTA/HAp composite as a biocompatible and osteoinductive composite for CSBD repair, presenting enhanced mechanical strength, bioactivity, and osteoconductivity.

## Introduction

Bone regeneration in critical-sized lesions constitutes a major difficulty in orthopaedic surgery and traumatology. Annually, over 150 million new fractures occur globally, and the management of considerable bone loss surpassing 2 cm or 50% of bone circumference is a huge healthcare challenge, as natural healing is unattainable in such instances, resulting in non-union^1^. The main gold standard for CSBD treatment is autologous or allograft bone transplantation which displays significant limitations despite its osteogenic and osteoconductive features^2^. Significant side effects as donor site morbidity and inadequate bone accessibility for locating, increased the risk of infection, and possible surgical site complications^3^. Moreover, autografts display upsetting outcomes, with an 8% possibility of infection recurrence and a 3% amputation rate^4^. Allograft procedures, even though extenuating donor site complications, display concerning consequences with a 29.8% structural failure rate and a 25.5% non-union rate^5^. Additional worries include the opportunity of disease transmission, even though strict screening, with recognized examples of viral infections such as human immunodeficiency virus (HIV) (1:1.6 million risk rate), Hepatitis B virus (HBV), and Hepatitis C virus (HCV). The decontamination measures required for allografts significantly lessen their mechanical characteristics and osteoinductive volume, compromising the strength of the grafted bone by coarsely 50%^6^. To address these limitations, innovative biomaterials are being developed to improve bone formation. Effective bone formation procedures necessitate an osteoconductive composite with appropriate mechanical stability with an osteoinductive stimulus to induce osteogenesis and the capacity to permit osseointegration and vascularity^7^. The current study concentrates on a novel bioactive composite that comprises nGO, MTA, and HAp. The characteristic construction of this composite influences the unique characters of each component to generate a synergetic effect that enhances bone regeneration. The integration of Zinc oxide (ZnO) nanoparticles conveys notable biological characters, enhancing the metabolic activity of the fibroblasts and the endothelial cells, both of them are crucial for effective implant incorporation^8^. This kind of composite displays a significant upgrading in cell proliferation, differentiation, and osseous formation^9^. The nGO sheets afford an excellent mechanical characteristic causing enhancement of the cellular responsiveness^10^. The material’s distinguishing molecular configuration which facilitates the three-dimensional composites with enhancement of the electrical conductivity, even though its wide-ranging precise surface area obviously augments the cell adhesion and osteogenic activity^10^. HA promises higher biocompatibility and osteoconductive characteristics, enabling osteoblast proliferation and osteocalcin formation^11^. Its chemical similarity to natural bone minerals enhances the direct adhesion to bone tissue, abolishing the formation of intermediate fibrous layers^12^. This nGO/MTA/HAp composite was designed to engage numerous corresponding signaling pathways vital for bone regeneration. The Wnt/β-catenin pathway is vital for controlling the differentiation of mesenchymal stem cells into osteoblasts and suppressing their differentiation into adipogenic and chondrogenic lineages^13^. The Bone morphogenetic protein (BMP) system regulates bone formation via both Smad-dependent and Mitogen-activated protein kinase (MAPK) signaling cascades^14^. Vascular endothelial growth factor (VEGF) signaling is crucial for connecting angiogenesis with osteogenesis, and sustaining optimal VEGF levels is vital for sufficient vascular invasion and improved bone production^15^. Notch signaling governs bone metabolism and vascular development, facilitating osteoblast differentiation and controlling bone resorption through the production of osteoprotegerin^16^. The current advances in biomaterials have displayed many strategies to improve bone regeneration, as the utilization of bioactive ceramics and smart stimuli-responsive materials^17^. For example, hydroxyapatite-based composites are well known for their exceptional osteogenesis and osseointegration nevertheless they suffer from a low rate of degradation with poor biomechanical characters^18^. Tricalcium phosphate, one more bioactive ceramic that also offers an excellent bioactivity with biocompatibility but has a high solubility and faster degradation rates, which can disturb osseointegration^19^. The integration of nanoparticles, as ZnO, with MTA has been shown to improve the compressive strength and bioactivity^20^. The current research aligns with current healthcare requirements and technological advancements in biomaterials. This study was conducted through in vivo experiments to assess the composite’s effectiveness in encouraging bone regeneration, assessing its mechanical properties, and examining its biological response in critical-sized defect models. The findings will contribute significantly to addressing the pressing need for effective bone healing solutions while establishing local expertise in advanced biomaterials development.

## Materials and methods

### Synthesis of graphene oxide nanosheets

nGO was produced with a modified Hummers process^21^. Two grams of graphite powder were combined with 30 milliliters of 98% sulfuric acid while being stirred vigorously for one hour. Potassium permanganate (6 g) was gradually added while the temperature was maintained at 35 °C using an ice bath. The solution was then diluted with distilled water and treated with hydrogen peroxide. After filtering and washing with hydrochloric acid, the precipitate was neutralised and sonicated for 90 minutes, yielding a homogenous graphene oxide solution that was subsequently dried at 60°C^22^. Sonication was shown to be necessary for attaining uniform dispersion and limiting nGO sheet aggregation.

### Procurement of hydroxyapatite (HA) and mineral trioxide aggregate

HAp was purchased from Sigma-Aldrich® (St. Louis, MO, USA), whereas the MTA was obtained from CERKAMED® (Stalowa Wola, Poland).

### Production of nGO/MTA/HAp composite

The nGO/MTA/HAp composite was synthesized by mixing and drying liquids. In summary, 0.50 g of graphene oxide nanosheets and 0.1 g of MTA were combined with 50 mL of 96% ethanol and sonicated for 1 hour to ensure equal dispersion of nGO. Following that, 1 g of HAp was added to the solution, which was sonicated for an additional 2 hours to improve homogeneity. This mixture was swirled for roughly 24 hours at room temperature to improve the interaction of all the components. The resulting composite was centrifuged, washed often with ethanol to remove any remaining reactants, and dried under a vacuum at 60 °C for 24 hours. The resultant composite had a weight ratio of around nGO: MTA: HAp = 0.5:0.1:1. The prolonged stirring and sonication procedures verified the uniform distribution of components, which is critical for the composite’s bioactivity and mechanical strength. This composite fabrication method-solution mixing coupled with extended sonication-was chosen based on evidence from recent literature indicating that such protocols ensure homogeneous dispersion of nGO within the mineral matrix, which is vital for achieving enhanced mechanical properties and bioactivity. Uniform distribution of nGO, MTA, and HAp maximizes the synergistic effects of each component, as supported by studies demonstrating improved osteogenic potential and mechanical strength in similarly prepared composites. The rationale for this method is to promote optimal interaction among all phases, facilitating effective cell adhesion, proliferation, and bone regeneration^23^**.**

### Characterization of nGO/MTA/HAp composite

The nGO/MTA/HAp composite was characterized using X-ray Diffraction (XRD), Scanning Electron Microscopy (SEM), and Fourier Transform Infrared Spectroscopy (FTIR) to establish its composition and structural features.

#### X-ray diffraction (XRD)

XRD measurements were performed using Cu Kα radiation (λ = 1.54186 Å) on a Philips PW1710 X-ray diffractometer. The XRD patterns were recorded from 10 ≤ 2θ ≤ 70°, with a step size of 0.020° 2θ and a counting time of 10 seconds per step. This range allowed for the identification of crystalline phases in HAp and MTA.

#### Scanning electron microscopy (SEM)

SEM images were obtained using a ZEISS FE-SEM ULTRA Plus microscope (equipped with an EDX analyzer) with a Philips CM20 microscope, operating at an accelerating voltage of 200 kV. SEM provided insights into the morphology and porosity of the composite, which are crucial for cell adhesion and proliferation.

#### Fourier transform infrared spectroscopy (FTIR) 

FTIR spectroscopy was carried out using a Nicolet 6700 spectrophotometer to identify the functional groups on the nanoparticle surface. FTIR analysis confirmed the presence of characteristic peaks for HAp, MTA, and nGO, validating the composite’s composition.

### In vitro degradation analysis

#### Sample preparation

Composite specimens (10 mm × 10 mm × 2 mm) were immersed in 50 mL of phosphate-buffered saline (PBS, pH 7.4) and incubated at 37 °C under gentle agitation (100 rpm) for 28 days. PBS was refreshed every 72 hours to maintain ion concentration and pH stability.

#### Degradation assessment

##### Mass loss measurement

Samples were rinsed with deionized water, dried at 60 °C for 24 hours, and weighed (analytical balance, ±0.1 mg) at predefined intervals (Days 7, 14, 21, 28). Mass loss (%) was calculated as:

1 $$\text{Mass loss}\left(\%\right)=\left(\frac{Wo-Wt}{W0}\right)\times 100$$

Where W_0_ = initial dry weight, W_t_ = dry weight at time t.

##### pH monitoring

The pH of the PBS solution was measured daily using a calibrated pH meter (Mettler Toledo, ±0.01 accuracy) to assess acidic/byproduct release.

##### Morphological analysis

Post-degradation samples were analyzed via SEM (JEOL JSM-IT500, 10 kV) to evaluate surface erosion, pore formation, and structural integrity.

##### Control

PBS without samples was incubated under identical conditions to account for environmental pH fluctuations.

### Animals

The study involved 24 healthy male Wistar rats (3-4 months old, weighing 260–320 g) obtained from an animal facility at the Faculty of Veterinary Medicine, Cairo University, Egypt.

They were kept in a controlled environment with a 12-hour light/dark cycle and had access to food and water ad libitum. The rats were randomly divided into two groups (n=12 each) based on the treatment administered:

- Group A: A critical-sized defect was surgically produced, and rats were used as a control group with no treatment.
- Group B: A critical-sized defect was surgically produced, and rats were treated with the nGO/MTA/HAp Composite implantation.

All methods were performed following the relevant guidelines and regulations. The work has been reported in line with the ARRIVE guidelines 2.0.

### Ethical statement

All methods were performed in accordance with the relevant guidelines and regulations. Surgical procedures were approved by the Institutional Animal Care and Use Committee, Cairo University, in May 2024, with the number CU II/F/23/24. This study complied with the ARRIVE guidelines during the experiment. The animals were monitored and maintained under standard conditions for one week before surgery to ensure proper acclimatization.

### Surgical procedures

**Fig. 1:**
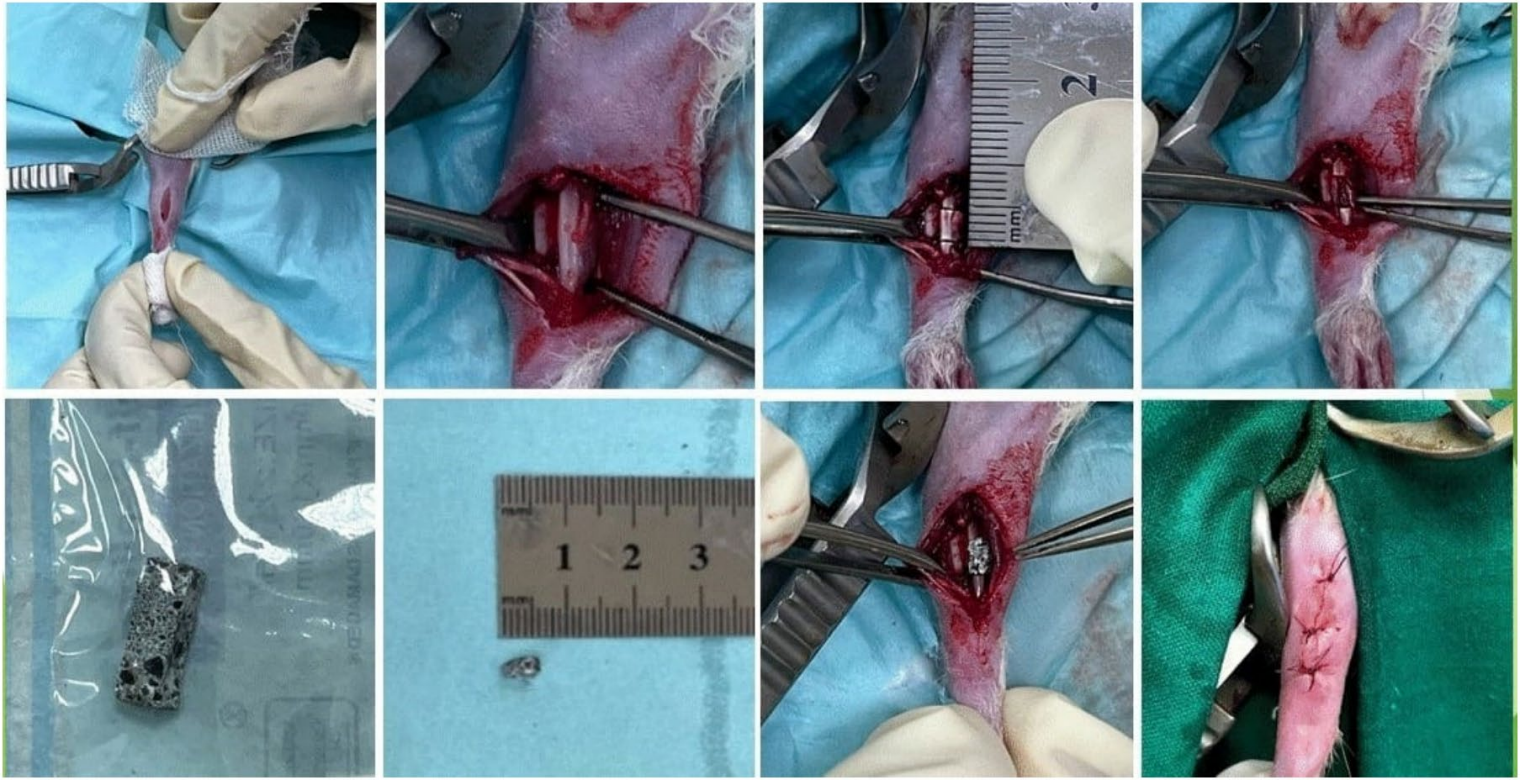
showing the surgical procedures for creating the bone defect and the implantation of the composite within the defect.

The Institutional Animal Care and Use Committee at Cairo University (Cairo, Egypt) approved the study methods under the designation CU II/F/23/24. The rats were anesthetized with a mixture of xylazine (10 mg/kg) (Xyla-Ject® 2% ADWIA Co., A.R.E.) and ketamine (70 mg/kg) (Ketamar® 5% Sol. Amoun Co. A.R.E) given intraperitoneally. To achieve analgesia, 1 mg/kg of meloxicam (Boehringer Ingelheim) was administered subcutaneously prior to the incision. A lateral incision was performed on the right radius, and the subcutaneous tissues and muscles were meticulously removed to reveal the radius. An electrical bone saw was employed to generate a 5-mm-long bone defect, and the periosteum was excised from the margins to inhibit ossification^24^. The nGO/MTA/HAp composite was molded into a cylindrical configuration corresponding to the defect dimensions (5mm in length, about 2 mm in diameter) before implantation. The composite material was then implanted without fixation in the treated group, whereas the control group received no therapy. Subsequently, the muscle fascia was sutured, and the incision was sealed (Fig. 1). Post-surgery, the rats received 20,000 U/d of gentamicin (gentamicin®40mgAlexandria Co., A.R.E.) via intramuscular injection for three days to prevent infections. They were housed separately to allow careful monitoring of recovery.

### Post-operative follow-up evaluation

#### Clinical examination

Post-operative evaluation involved daily and weekly monitoring of vital signs (body temperature, respiratory rate, weight, and overall activity). Daily inspection of the surgical wound site for signs of infection, seroma formation, local hyperthermia, and other complications.

#### Radiographic assessments

Mediolateral radiographs were taken of the operated radius using an X-ray apparatus (Siemens 300) at 45 kV, 30 mAs, and 80 cm FFD before surgical operation and immediately after the operation (zero-day) and at 2, 4, and 8 weeks postoperative. The bone healing was assessed in terms of radiographic density, the degree of implant incorporation at the implantation site, and the new bone deposition filling of the bone defect^25^**.** Radiographic evaluation of both groups (A&B) was assessed by two expert radiographers blind to the study design. The Lane and Sandhu scoring system was employed to quantify bone healing on radiographs.

#### Computed tomography assessments

The treated radius was imaged with a spiral-CT. Multidetector CT (Optima GE520) −16 slices at 2-, 4-, and 8-weeks post-operative utilizing a middle-frequency kernel and 0.6-mm-thick axial images. The obtained images were reconstructed in three dimensions (3D) using the RadiAnt DICOM image processing software (Medixant Co, Poznan, Poland)^26^**.** 3D reconstruction allowed for detailed visualization of bone formation and defect closure.

### Histopathological examination

Rats of each group were euthanized at 2, 4, and 8 weeks postoperatively using cervical dislocation under anesthesia. After decalcification of the bone samples in 10% Ethylenediaminetetraacetic acid (EDTA) (pH 7.4) for 35 days, all samples were embedded in paraffin wax and used for sectioning. The 5-μm tissue sections from the paraffin-embedded samples were then stained with hematoxylin and eosin (H&E)^27^. Histological analysis provided insights into composite integration and new bone formation.

#### Calculation methodology to assess inflammation, fibrous tissue, and bone formation

The percentages of inflammation, fibrous tissue formation, and bone formation were determined using a semi-quantitative scoring system combined with digital image analysis, as outlined below:

##### Semi-quantitative scoring (Manual assessment)

A trained pathologist evaluated histopathological slides using a predefined ordinal scale adapted from established protocols. For each parameter (inflammation, fibrous tissue, bone formation), the pathologist assigned a score based on the proportion of the region of interest (ROI) occupied by the tissue type (Table 1):

**Table 1:** Semi-quantitative scoring.

Score	Percentage range	Definition
1	<5%	Rare/negligible presence
2	6–40%	Multifocal or moderate presence
3	41–80%	Coalescing or extensive presence
4	>80%	Diffuse or near-total presence

These scores were averaged across multiple fields (e.g., 10 random 400× fields per sample) to derive the final percentage estimates.

##### Digital image analysis (Automated quantification)

1. Software: ImageJ (v1.53) were used to analyze stained histological sections.
2. Protocol:

1. ROI selection: A representative region of the composite-tissue interface was selected.
2. Thresholding: Tissue types were differentiated by color (e.g., inflammation: blue nuclei; fibrous tissue: pink collagen; bone: purple mineralization).

3. Area calculation: The area occupied by each tissue type was measured and expressed as a percentage of the total ROI area:
  2 $${\text{Percentage}}\;\% \; = \;\left( {\frac{Area\;of\;tissue\;type}{{Total\;ROI\;area}}} \right) \times 100$$

#### Interpretation of 100%

1. **100% inflammation:** The entire ROI is occupied by inflammatory cells (e.g., lymphocytes, macrophages), with no bone or fibrous tissue present.
2. **100% fibrous tissue:** The ROI is entirely composed of collagen-rich connective tissue, with no bone or inflammation.
3. **100% bone formation:** The ROI is fully mineralized bone, with no residual fibrous tissue or inflammation.

These percentages are mutually exclusive within a defined ROI, and their sum typically approaches 100% (e.g., 60% bone, 30% fibrous tissue, 10%)^28^**.**

### Statistical analysis

Data were analyzed using one-way ANOVA, with statistical significance set at p ≤ 0.05. Results were reported as means, medians, and standard deviations. Statistical analysis was conducted using SPSS software (version 20.0; IBM Corp., Armonk, NY, USA). ANOVA allowed for the comparison of means among groups to identify significant differences in bone healing and biochemical parameters.

## Results

### Characterization of nGO/MTA/HAp composite

#### X-ray diffraction patterns (XRD)

**Fig. 2:**
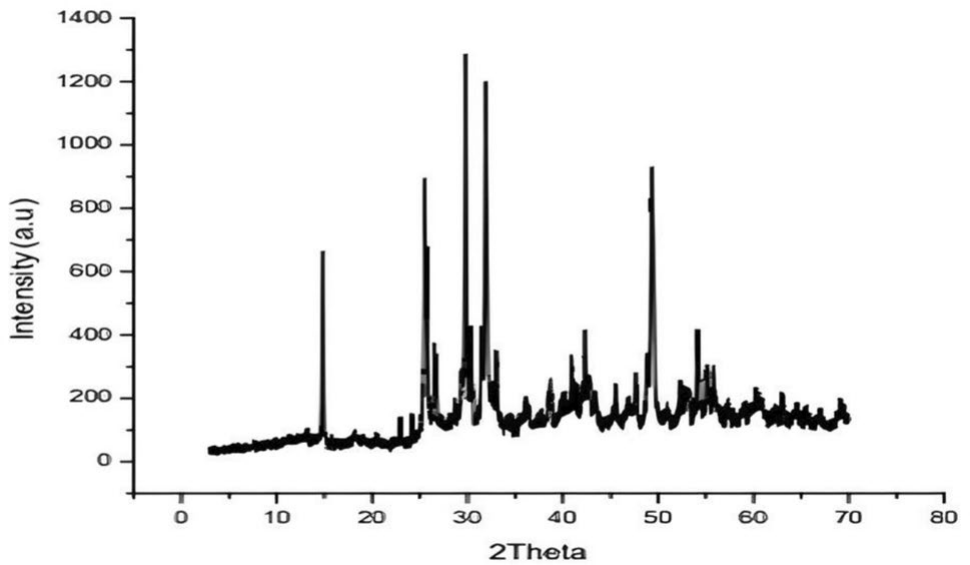
XRD of HAp/MTA/nGO composite.

The XRD patterns of the nano-GO/MTA/HAp composite confirmed the presence of HAp, MTA, and nGO phases. The X-ray diffraction patterns (XRD) of nGO/MTA/HAp composite were depicted in (Fig. 2).

The X-ray diffractogram of HAp present in the composite was in good agreement with the standard values given in the JCPDS data (09-0432). Notable High-intensity peaks at 2θ values around 25.54°, 29.80°, 31.93°, 32.1°, 36.08, 38.68° and 40.87° are associated with the (002), (210), (211), (112), (300), (202) and (222) crystallographic planes, respectively, confirming the specific crystallinity of HAp. These sharp and strong peaks specify the high crystallinity of HAp, which is vital for its bioactivity and mechanical strength.

XRD of MTA reveals peaks corresponding to these calcium silicate phases, indicating its hydraulic cement nature. Overlapping peaks at 25–34° are observed suggesting a combination of HAp and MTA phases. Sharp and intense peaks suggest the presence of crystalline phases (HAp and MTA). nGO exhibits a peak at 2θ = 14.83°attributed to the (001) reflection of oxidized graphene layers (Table 2). Extra peaks at higher angles (40–50°) could confirm the presence of bismuth oxide, a component of MTA and other calcium silicate phases.

3 $${\text{D}} = \frac{{{\text{K}}\uplambda }}{{\upbeta \cos\uptheta }}$$

**Table 2:** Summarizes the calculated crystallite sizes, d-spacing values, and phases for each peak.

Peak (2θ)	d-spacing (Å)	FWHM (radians)	Crystallite size (nm)	Phase
25.54	3.49	0.10	14.86	Hydroxyapatite
29.80	3.00	0.12	12.50	Hydroxyapatite
31.93	2.80	0.08	18.84	Hydroxyapatite
32.10	2.79	0.09	16.76	Hydroxyapatite
36.08	2.49	0.11	13.86	Hydroxyapatite
38.68	2.33	0.10	15.36	Hydroxyapatite
40.87	2.21	0.13	11.90	Hydroxyapatite
14.83	5.97	0.15	9.74	Graphene oxide

The crystallite size (DDD) of HAp and nGO was calculated using the Scherrer equation (3):

Where K=0.94 is the Scherrer constant, λ=1.54186 A˚ is the X-ray wavelength, β is the full width at half maximum (FWHM) in radians, and θ is the Bragg angle in radians.

The results confirm that the composite contains highly crystalline HAp and MTA phases, along with oxidized graphene layers.

#### SEM, EDS and elemental mapping of nGO/MTA/HAp composite

**Fig. 3:**
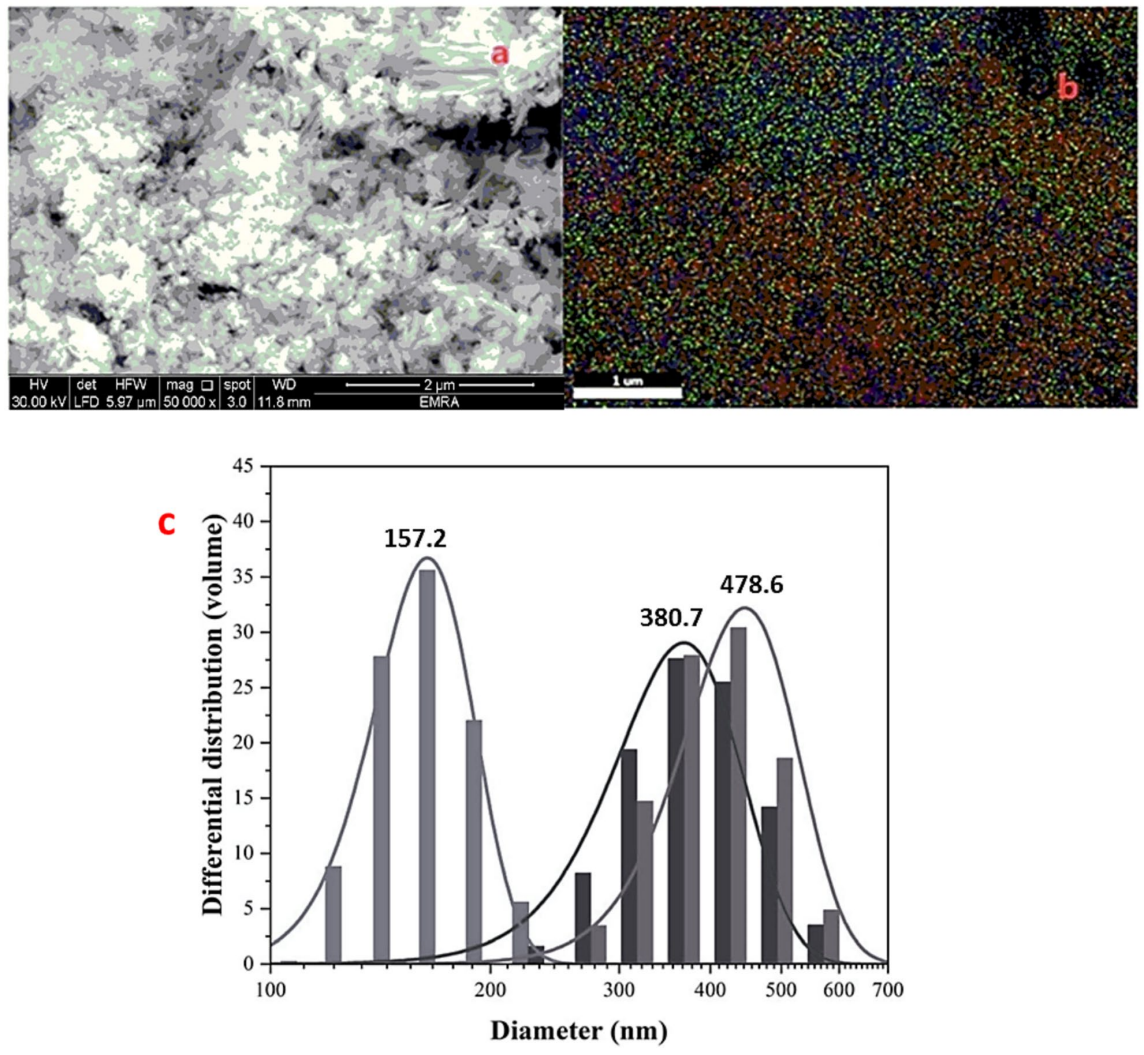
(**a**) SEM of nGO/MTA/HAp composite, (**b**) Elemental mapping of the composite. (**c**) Volume basis analysis

**Fig. 4:**
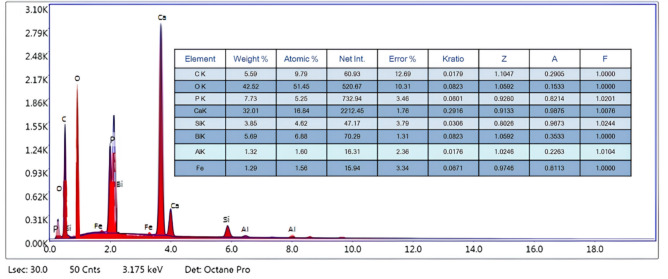
EDS spectrum of nGO/MTA/HAp composite.

**Fig. 5:**
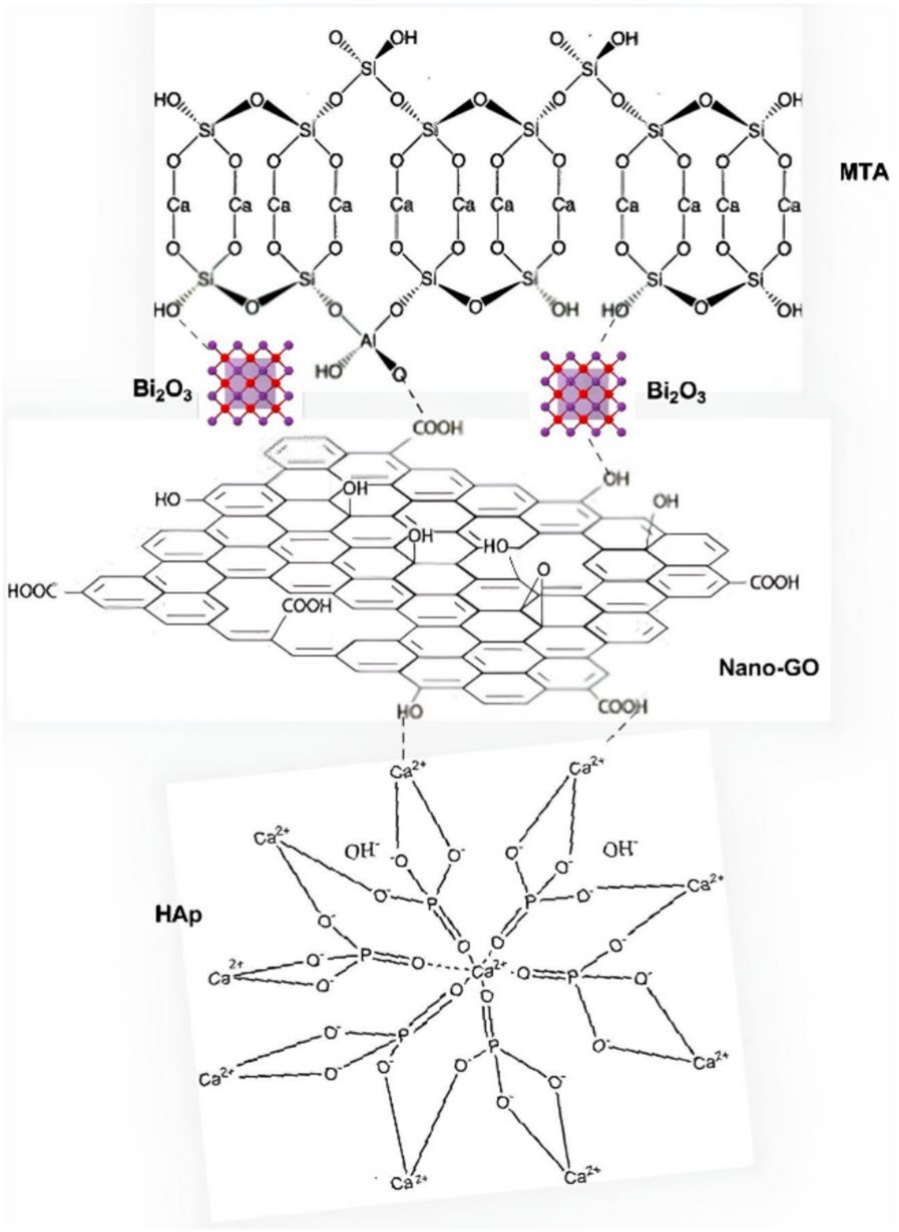
Chemical compositions of nGO/MTA/HAp composite. Generated by PerkinElmer ChemDraw Professional, version. 15.0.

The SEM image (Fig. 3.a.) showed an agglomerated, “fluffy” network of particles of HAp and MTA that are uniformly distributed over or interspersed with nGO. The image displays a characteristic nGO/MTA/HAp composite with rod-like HAp crystallites aggregated with the porous MTA structures into dense clusters, likely anchored on or interspersed with thin graphene oxide layers. Graphene oxide nanosheets can appear as wrinkled or thin layered regions, so the HAp crystals seem to dominate the view. The overall morphology is porous and rough, which can be beneficial in many bone-related or composites applications. Energy-dispersive X-ray spectroscopy (EDS) elemental mapping (Fig.3. b.) illustrates a uniform and favorable distribution throughout the composite. Volume basis analysis was used to assess the particle diameter (Fig. 3.c.). The average particle sizes were found to be 157.2 nm, 380.7 nm, and 478.6 nm, for the nGO/MTA/HAp composite. It was investigated that a narrow size distribution was observed that means the powder blends with larger particle size in ethanol solution, resulting in a decrease in the heterogeneity of the particles, and particle agglomeration was favored. Results showed that prepared materials were in the sub-micrometric/micrometric range (150 to 600 nm) with maximum particle size distributions not exceeding 1000 nm. This finding underscores the fact that larger particles tend to flow more easily than smaller particles, which affects the proximity of the particle cluster. EDS spectrum c confirms the presence of elements C, O, P, Ca, Si, Bi, Al and Fe elements (5.59 wt % C, 42.52 wt % O, 7.73 wt% P, 32.01 wt% Ca, 3.85wt% Si, 6.69 wt% Bi, 1.32 wt% Al, and 1.29 wt% Fe), without impurities (Fig. 4). The results confirmed on the preparation of nGO/MTA/HAp composite, where MTA is a calcium silicate-based bioactive material that is mainly comprised of Portland cement (tricalcium silicate (3CaO.SiO_2_), dicalcium silicate (2CaO.SiO_2_), tricalcium aluminate (3CaO.Al_2_O_3_), and a tetra-calcium aluminoferrite (4CaO.Al_2_O_3_Fe_2_O_3_)], and bismuth oxide. In addition, the HAp is a naturally occurring mineral form of calcium apatite, characterized by the chemical formula Ca_5_(PO_4_)_3_(OH). According to the results, the prepared material nGO/MTA/HAp composite can be expressed as the following chemical compositions (Fig. 5).

### Quantitative analysis of pore size and porosity

ImageJ software was used to analyze the SEM images quantitatively. The pore size distribution and porosity percentage of the composite were calculated based on three SEM images, with five measurements per image. The results are summarized as follows:

$${\text{The}}\;{\text{Mean}}\;{\text{Pore}}\;{\text{Size}}:{ 3}00 \pm {5}0\,\mu {\text{m}}\;{\text{while}}\;{\text{the}}\;{\text{Porosity}}\;{\text{Percentage}}:{ 65} \pm {5}\%$$

These values confirm that the composite has an interconnected porous structure suitable for bone regeneration, as pore sizes between 300–400 µm are optimal for vascularization and osteogenesis.

#### FTIR of nGO/MTA/HAp composite

**Fig. 6:**
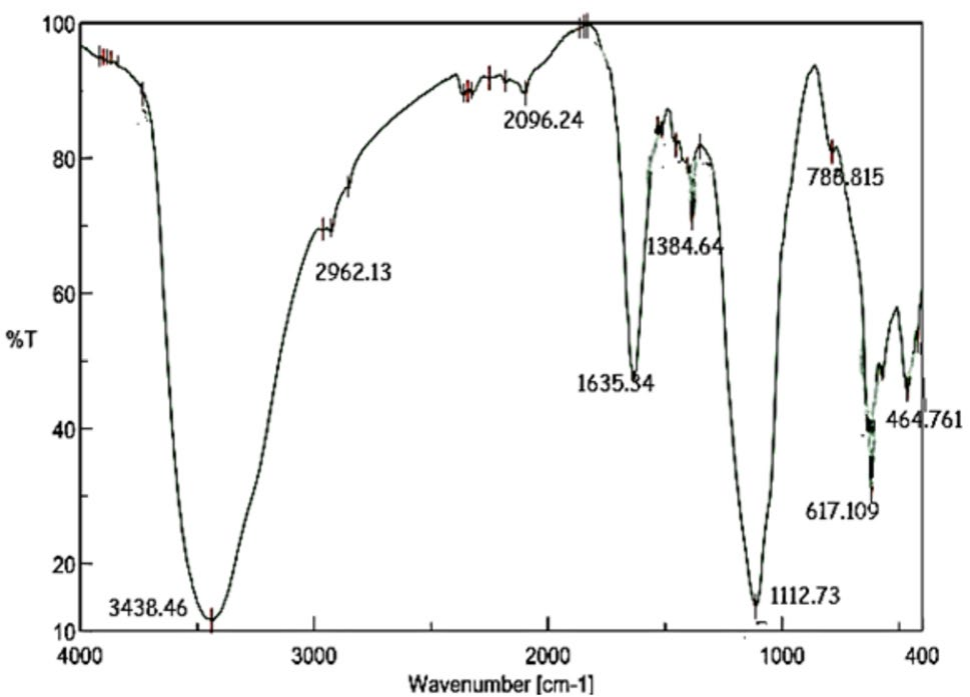
FTIR pattern of (HAp/MTA/nGO) composite.

FTIR spectrum in (Fig. 6) shows a peak at 3438.46 cm⁻^1^ corresponds to the O-H stretching vibrations of the hydroxyl groups that are found in both HAp and MTA. The peak observed at 2962.13 cm⁻^1^ represents the C-H stretching vibrations of nGO, weak peak at 2096.24 cm⁻^1^ can be attributed to the (C-O-C) functional group in graphene oxide. The peak at 1635.34 cm⁻^1^ likely corresponds to the C=O stretching vibration from graphene oxide (GO). It is also noticed a peak at 1384 cm⁻^1^ that is likely attributed to carbonate groups (CO₃^2^⁻), which are found in MTA. The 1112.73 cm⁻^1^ peak indicates phosphate stretching (PO₄^3^⁻), which is a characteristic feature of HAp. Strong Peak at 617.11 cm⁻^1^ is associated with phosphate (PO₄^3^⁻) bending vibrations (P-O) of HAp, while the peak at 464 cm⁻^1^ confirms the presence of HAp through P-O bending.

These FTIR findings confirm the presence of HAp, MTA, and nGO phases within the composite, validating its composition.

### In vitro degradation study

An in vitro degradation study was performed in a phosphate-buffered saline (PBS) solution at pH 7.4 and 37 °C to determine the stability and possible toxicity of the nGO/MTA/HAp combination. The composite samples were submerged in PBS for 28 days and measured at 7, 14, 21, and 28 days.

#### Weight loss and surface morphology changes

The weight loss of the composite was monitored at each interval to calculate its deterioration rate. The study found that weight loss gradually increased over time, reaching 5.2 ± 1.1% on day 28.

This indicates that the composite undergoes slow and controlled degradation, which is favorable for maintaining composite integrity during the initial stages of bone healing (Table 3).

**Table 3:** The mean (±SD) values for summarizes the weight loss of the composite measured at each interval to determine its degradation rate.

Time (days)	Weight loss (%)	SEM observations
7	1.2 ± 0.3	Minimal surface change
14	2.5 ± 0.5	Initial surface erosion
21	3.8 ± 0.7	Visible surface degradation
28	5.2 ± 1.1	Significant surface alteration

#### Surface morphology changes (SEM analysis)

- At Day 7: SEM images revealed minimal surface erosion, with the composite retaining its porous architecture.
- Day 14: Initial signs of surface roughness and microporosity were observed, suggesting early-stage degradation.
- Day 21: Increased porosity and surface erosion, though the composite maintained interconnected pores critical for cell infiltration.
- Day 28: Significant surface alterations, including 30–50 µm pore enlargement and localized dissolution of MTA/HAp particles, while graphene oxide nanosheets remained structurally intact.
- Key Observation: The composite’s degradation occurred preferentially at the MTA/HAp interfaces, while nGO provided structural reinforcement, delaying catastrophic failure.

#### pH stability

The pH of the PBS solution was monitored throughout the study to evaluate whether the composite’s degradation affected the surrounding environment. The pH remained relatively stable, with minor fluctuations around the initial value of 7.4. This stability suggests that the degradation process does not significantly alter the pH, which is critical for maintaining biocompatibility and preventing adverse effects on surrounding tissues.

Baseline pH: 7.4 ± 0.1.

Post-degradation pH: At - 7 days: 7.38 ± 0.05. - 14 days: 7.35 ± 0.07. - 21 days: 7.32 ± 0.06. - 28 days: 7.30 ± 0.08.

### Post-operative evaluation of the surgical wound site

Animals in both groups exhibited complete wound closure within seven days. This healing was observed without any significant complications in either group.

Only one rat showed post-operative minor swelling that resolved spontaneously within 48 hours. No infections or wound dehiscence were noted in any animals suggesting that both groups tolerated the surgical procedure well, with no adverse effects attributed to the nGO/MTA/HAp composite.

### Radiographic observations and computed tomography

#### Radiographic scoring

Quantitative radiographic analysis using the Lane and Sandhu scoring system revealed significant differences between the A&B groups. The mean scores for each group at each time point are summarized in (Table 4).

**Table 4:** The mean (±SD) values and p-value for Quantitative radiographic analysis of both treated and untreated groups using the Lane and Sandhu scoring system.

Parameter	Group	2 weeks	4 weeks	8 weeks	p-value
Lane and Sandhu Score	A group	1.5 ± 0.2	2.0 ± 0.3	2.5 ± 0.4	<0.001
	B group	3.0 ± 0.3	4.0 ± 0.4	5.0 ± 0.5	

The treated group B consistently showed higher scores, indicating enhanced bone healing compared to the control group.

### Radiographic observations

#### Control group (A group)

**Fig. 7:**
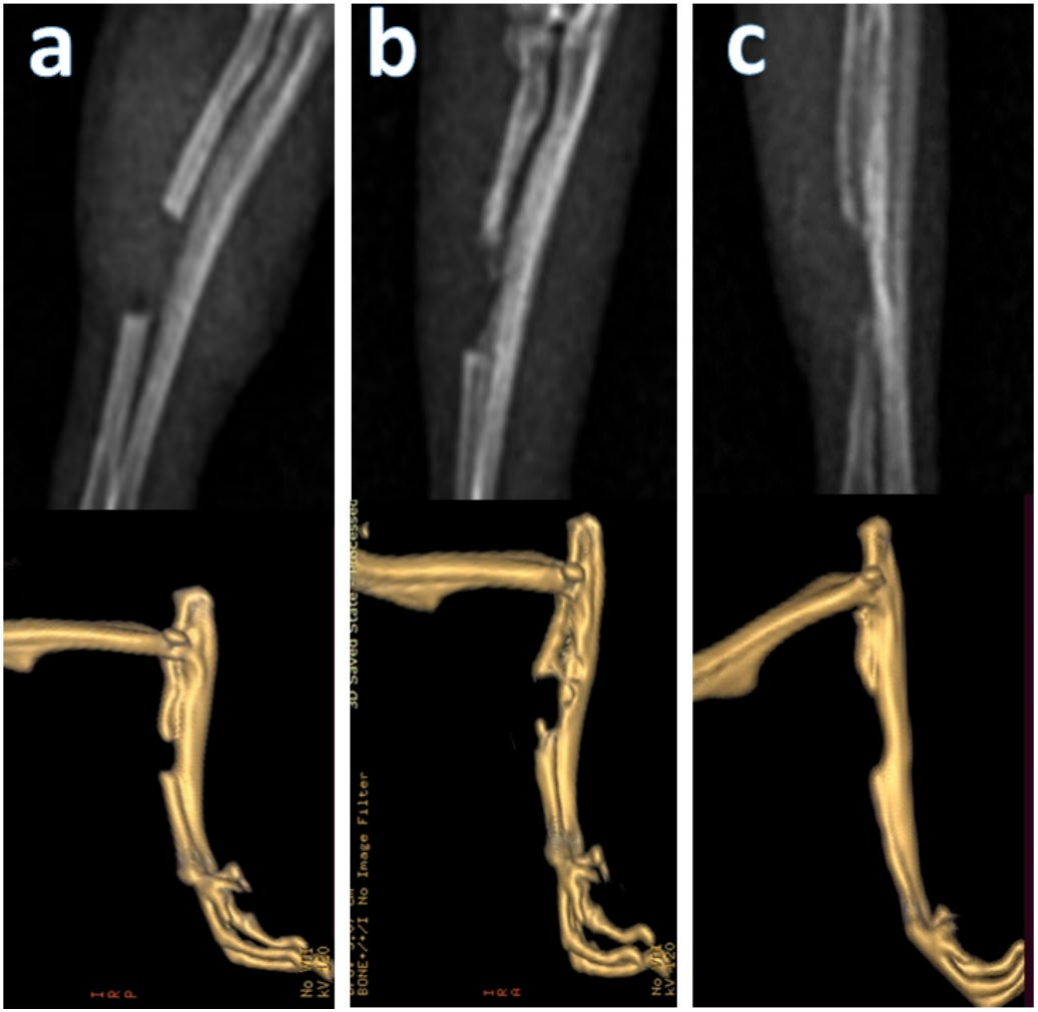
The radiographic and computed tomographic scans of the bone defect of the control group 2 weeks (**a**), 4 weeks (**b**), and 8 weeks (**c**) postoperatively.

At 2 and 4 weeks, no radiographic changes were observed, with the defect appearing transparent and clearly visible. By 8 weeks, a radiopaque area was seen at the distal cortex without signs of remodeling, and the defect size remained unchanged (Fig. 7).

#### Treated group (B group)

**Fig. 8:**
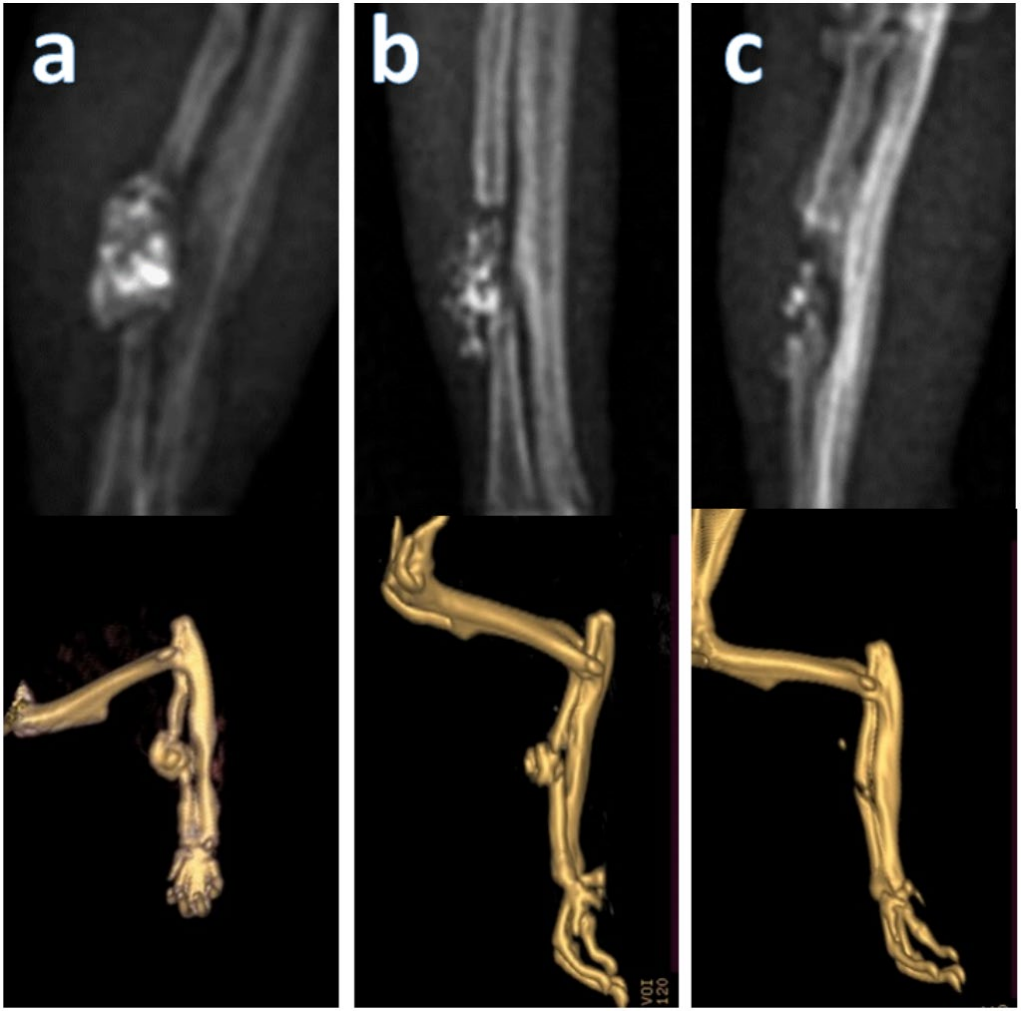
The radiographic and computed tomographic scans of the bone defect of the composite group 2 weeks (**a**), 4 weeks (**b**), and 8 weeks (**c**) postoperatively.

At 2 weeks, the implant remained well-aligned with the fracture ends, exhibiting a clear pore structure with a visible defect. By 4 weeks, the implant was partially absorbed, shortened, and narrowed, with the callus wrapping around the material. At 8 weeks, there was a marked increase in newly formed bone, uniting the bone ends and appearing more radiopaque (Fig. 8).

### Computed tomography (CT) analysis

CT scans were analyzed to quantify bone volume (BV), bone mineral density (BMD), and trabecular thickness (Tb. Th) at each time point using appropriate image segmentation software (Table 5).

**Table 5:** The mean (±SD) values and p-value for Quantitative computed tomographic analysis of both treated and untreated groups appropriate image segmentation software.

Parameter	Group	2 Weeks	4 Weeks	8 Weeks	p-value
Bone volume (BV) mm^3^	A Group	15 ± 2	20 ± 3	25 ± 4	<0.01
	B group	50 ± 5	70 ± 6	85 ± 7	
Bone mineral density (BMD) g/cm^3^	A Group	0.4 ± 0.05	0.5 ± 0.06	0.6 ± 0.07	<0.001
	B group	1.2 ± 0.1	1.5 ± 0.1	1.8 ± 0.2	
Trabecular thickness (Tb. Th) μm	A group	0.08 ± 0.01	0.10 ± 0.02	0.12 ± 0.03	<0.01
	B Group	0.25 ± 0.03	0.30 ± 0.04	0.35 ± 0.05	

Representative CT images are shown in Figs. 7 and 8 highlighting the progressive bone formation in the treated group compared to minimal changes in the control group.

#### Control group (A group)

CT scan of the control group at 2 weeks showed that the size of the defect (5mm) was clear. At 4 weeks the size of the defect remained clear and no significant changes were observed from the previous period. At 8 weeks no significant changes were observed from the previous period the defect remained clear. At 12 weeks showed a tapering was shown at the distal end of the bone defect (Fig. 7).

#### Treated group (B group)

CT scan of the composite group shows that at 2 weeks that the defect was filled with the composite material, which was well-aligned with the fracture ends. At 4 weeks. The defect implanted with composite showed new bone formation at the defect distal ends and the composite exhibited a partial absorption. At 8 weeks the defect implanted with composite showed a marked increase in the amount of newly formed bone appeared well in the defect area resulting in a significant reduction in bone defect size compared to the previous period and the control group also the composite appeared completely absorbed and degraded (Fig. 8). Statistical analysis using ANOVA revealed significant differences between the treated and control group for all parameters at each time point, confirming enhanced bone regeneration in the treated group.

### Histopathological evaluation for the composite

#### 2-Week critical bone defect and treated group

**Fig. 9:**
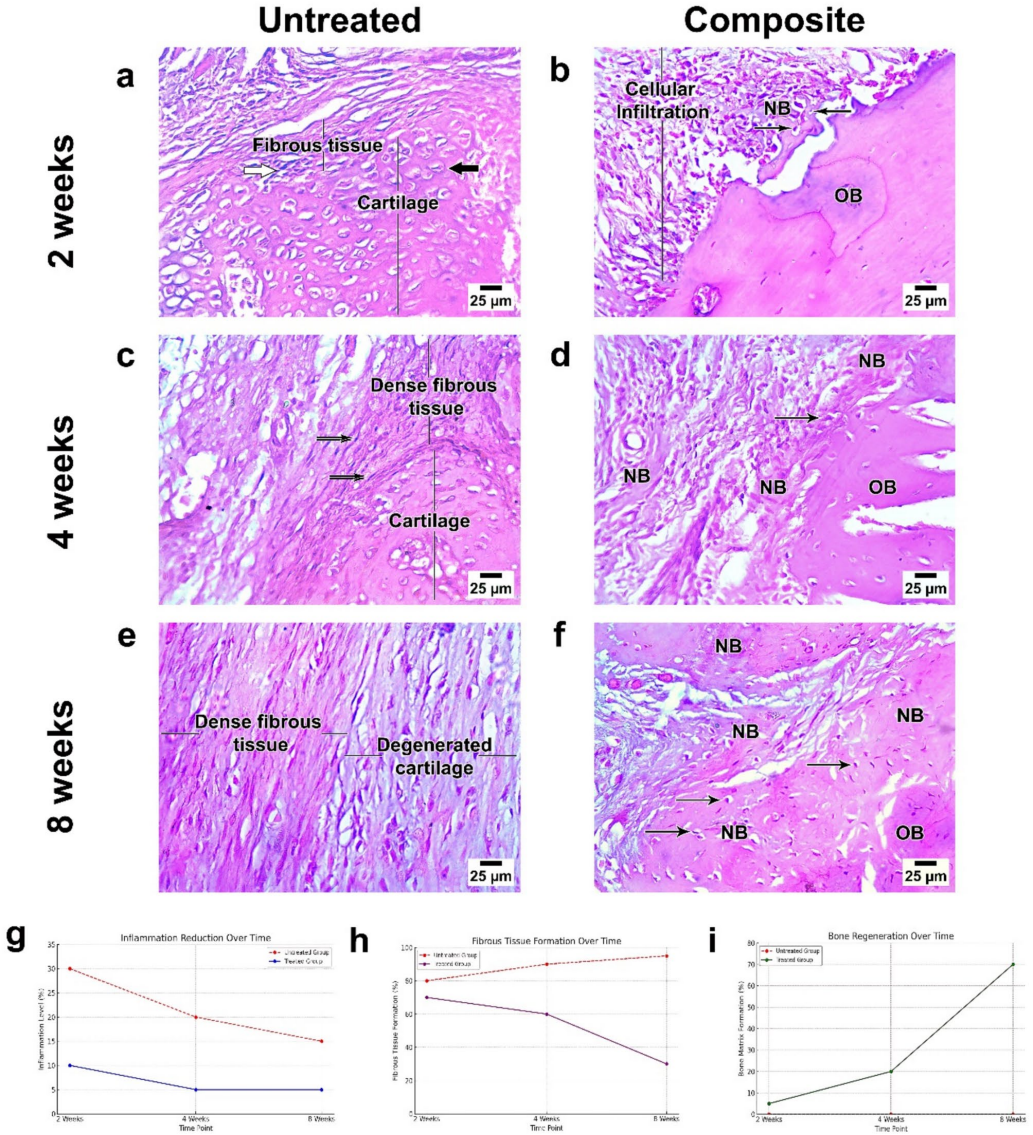
(**a**, **c**, **e**) shows a Histological section of a critical-sized bone defect showing a complete absence of bone tissue, necrosis at the defect margins, and limited fibrous tissue ingrowth. (**b**, **d**, **f**) Histological section of a bone defect treated with a nanoparticle composite, demonstrating composite integration, cellular infiltration, and new bone formation. (**g**, **h**, **i**) shows a Time-course analysis of histopathological tissue responses for inflammation, fibrous tissue formation and bone formation levels in treated and untreated groups throughout the different periods.

At two weeks post-injury, the untreated critical bone defect (Fig. 9 (a)) showed the formation of fibrocartilaginous tissue, characteristic of the early reparative phase. Chondrocytes were embedded within a cartilaginous matrix, and fibroblasts contributed to the surrounding fibrous tissue. This stage represented the initial stabilization of the fracture site through the formation of a soft callus, which is a critical step in the bone healing process. In contrast, the nanoparticle composite-treated group (Fig. 9 (b)) demonstrated early signs of composite integration with host tissue. The composite’s porous structure facilitated moderate cellular infiltration, primarily consisting of osteoblasts and fibroblasts, which are essential for initiating bone healing. Minimal inflammation was observed, indicating good biocompatibility of the composite material. This figure highlighted the composite’s ability to support bone regeneration by promoting tissue ingrowth and cellular activity.

#### 4-Week critical bone defect and treated group

At four weeks after injury, the untreated critical bone defect (Fig. 9 (c)) showed thick fibrous tissue with elongated fibroblast-like cells grouped in parallel bundles. There was no indication of new bone matrix development or osteoblast activity, indicating that the lesion was unable to repair naturally. The fibrous tissue indicated the body’s limited attempt to stabilise the deficiency in the absence of osteogenic activity.

In contrast, the composite-treated group (Fig. 9 (d)) exhibited encouraging signals of tissue integration and early bone repair. Cellular infiltration was noticeable, with fibroblast-like cells and early osteoblast development seen inside the composite material. The freshly produced bone matrix was apparent, but disorganised and lacking the mature lamellar structure found in normal bone. Tissue ingrowth into the composite’s porous structure suggested that the composite facilitated the initial stages of healing while minimizing inflammation.

#### 8-Week critical bone defect and treated group

At eight weeks post-injury, the untreated critical bone defect (Fig. 9 (e)) showed no bone tissue inside the defect region. The defect borders were characterized by shattered bone edges, necrosis, and inflammatory cell infiltration. Limited fibrous tissue ingrowth was observed at the periphery, but no significant bone regeneration was evident. The absence of osteoblasts and a new bone matrix confirmed the inability of the defect to heal spontaneously.

In contrast, the composite-treated group (Fig.9 (f)) demonstrated successful composite integration within the defect. The composite material appeared biocompatible, with no signs of adverse reactions or extensive inflammatory responses. Cellular infiltration was observed throughout the composite, with fibroblast-like cells and some evidence of early osteoblast differentiation. However, the newly formed bone matrix was limited and lacked the organized lamellar structure observed in the control group.

Histopathological tissue response evaluations for inflammation, fibrous tissue formation, and bone formation were summarized in (Tables 6,7,8). The results showed that a significant reduction in inflammation occurred throughout the study in the treated group compared to the untreated group (Fig. 9 (g)). Fibrous tissue formation dropped from 70% to 30% in the treated group while the untreated group showed persistent fibrous tissue formation reaching 95% at 8 weeks (Fig. 9 (h)). The untreated group exhibited no bone growth throughout the time of the study, while the treated group results show improved bone regeneration reached 70% by 8 weeks (Fig. 9 (i)).

**Table 6:** Evaluation of inflammation.

Time point	Untreated group	Treated group
2 Weeks	30% (moderate inflammation)	10% (minimal inflammation)
4 Weeks	20% (limited inflammation)	5% (minimal inflammation)
8 Weeks	15% (persistent inflammation)	5% (minimal inflammation)

**Table 7:** Fibrous tissue formation.

Time point	Untreated group	Treated group
2 Weeks	80% (dense fibrous tissue)	70% (fibrous tissue with composite)
4 Weeks	90% (predominant fibrous tissue)	60% (fibrous tissue with early bone matrix)
8 Weeks	95% (persistent fibrous tissue)	30% (reduced fibrous tissue with bone matrix)

**Table 8:** Bone formation.

Time point	Untreated group	Treated group
2 Weeks	0% (fibrocartilage)	5% (early bone matrix)
4 Weeks	0% (no bone matrix)	20% (disorganized bone matrix)
8 Weeks	0% (no bone regeneration)	70% (disorganized bone matrix)

## Discussion

In orthopaedic surgery, CSBDs and delayed or non-union fractures provide substantial hurdles since they cannot heal on their own without specific intervention^29^. Traditional bone grafts, which were formerly thought to be the best option for treating these kinds of problems, have several disadvantages. These include a significant likelihood of healing failure in big bone lesions, increased discomfort and morbidity, immunological rejection, and the requirement for donor site harvesting^30^. Additionally, these grafts sometimes need lengthy surgical procedures, which might result in problems and extended recovery periods^31^.

To overcome these limits, modern bone regeneration techniques primarily rely on physiologically active materials or osteoconductive composites. These efforts aim to speed bone healing by feeding cells capable of producing bone, either alone or in combination with bioactive chemicals^32^**.** The disadvantages of autografts, allografts, and xenografts can be addressed with osteoconductive composites, which provide a framework for cellular ingrowth and tissue integration^33,34^. By including bioactive components in these composites, osteogenic activity can be boosted, resulting in faster healing and better clinical outcomes^35^. Because of its osteoconductivity, HAp is commonly used in bone fillers and composites^36^, whereas MTA has high biological activity and is non-cytotoxic^37^. nGO has emerged as a potential addition to bone tissue engineering due to its ability to imitate bone stiffness and increase composite performance^38^. Furthermore, adding nGO to composites improves their mechanical strength and suitability for load-bearing applications. This enhancement could be determined according to the tensile strength of nGO. In the following table we examined the difference in tensile strength of nGO compared to the other three prepared composites: nGO: MTA: HAP (Table 9)^39^**.**

$${\text{Tensile}}\;{\text{strength}}\;{\text{formula}}:s = \, P/a$$

**Table 9:** Tensile strength of the prepared composite nGO: MTA: HAp.

Concentration of nGO in different composites	(nGO: MTA: Hap). Composite/ratio	Tensile strength^*^
nGO (100%)	1:0:0	2300 GPa
nGO (15.38%)	0.2:0.1:1	2550 GPa
nGO (31.25%)	0.5:0.1:1	3900 GPa
nGO (40.54%)	0.75:0.1:1	2830 GPa
nGO (47.62%)	1:0.1:1	2740 GPa

Where (**s)** is the tensile strength, **(P)** is the force required to break and **(**a**)** is the cross-sectional area.

Tensile Strength Formula: The formula is: s = P/a where s is the tensile strength P is the force required to break a is the cross-sectional area.

By combining the benefits of HAp, MTA, and nGO, the HAp/MTA/nGO composite provided improved osteoconductivity, osteointegration, and biocompatibility. By offering a favourable composite for cell infiltration and new bone development, this composite is intended to aid in bone repair. Compared to employing these materials alone, their combined impacts resulted in a better mechanical and biological quality. With contributions from all three components^40–42^. A critical aspect of this work was the selection of the composite fabrication protocol. For composite fabrication, a solution mixing protocol combined with extended sonication was adopted to ensure homogeneous dispersion of nGO within the MTA and HAp matrix. Literature supports that this approach is superior to simple mechanical mixing or dry blending, which often results in agglomeration of nGO and poor interfacial bonding. Homogeneous dispersion maximizes the synergistic effects of all composite components, resulting in enhanced mechanical strength, controlled degradation, and improved osteogenic potential. Polymeric and ceramic composite scaffolds fabricated with careful control over mixing and dispersion have been shown to outperform conventional materials in terms of bioactivity, biocompatibility, and mechanical properties, which are essential for successful bone tissue regeneration. Thus, the chosen synthesis and fabrication protocols align with current best practices and are justified by both their safety profile and their ability to yield composites with optimal physicochemical and biological performance^23^**.**

the HAp/MTA/nGO composite’s XRD pattern showed a complicated crystalline structure. The existence of crystalline HAp was confirmed by the good match between the diffraction peaks of HAp and the JCPDS data (09-0432). A distinct crystal structure was indicated by high-intensity peaks at particular 2θ \theta2θ values that matched different HAp crystallographic planes. In order to provide a robust foundation for bone regeneration, this crystallinity was essential^43^.

The MTA hydraulic cement nature inside the composite was confirmed by the presence of calcium silicate phases in the XRD pattern. The (001) reflection of oxidised graphene layers was identified as the source of a distinctive peak of nGO at 2θ=14.83∘2 \theta = 14.83^{\circ}2θ=14.83∘, confirming the presence of nGO in the composite^44^. The overlapping peaks in the 25−34∘25-34^{\circ}25−34∘ range indicated a tight link between the HAp and MTA phases. This suggested a synergistic effect, which might increase the mechanical and biological properties of the composite. The abrupt and forceful peaks demonstrated the composite’s high crystallinity, which is beneficial for maintaining structural integrity. Other peaks at higher angles corroborated the composite’s complex structure, most likely corresponding to bismuth oxide from MTA and other calcium silicate phases^45^.

The HAp/MTA/nGO composite’s shape and structure were revealed by the SEM picture, which showed an agglomerated,“fluffy”network of particles suggestive of a complicated three-dimensional structure. MTA created porous structures, whereas HAp crystallites showed up as rod-like structures. To promote cell attachment and proliferation, the components must be well integrated, as indicated by the uniform distribution of HAp and MTA particles. Though they might not have been as noticeable because of the predominance of HAp crystals, GO nanosheets were probably present as thin, wrinkled layers. For bone-related or composite applications, the composite’s general rough and porous shape is advantageous because it may encourage cell adherence and proliferation^46^. This shape promotes osteogenic activity, which makes it easier for the composite to integrate with the host tissue.

O-H stretching vibrations, which are present in both HAp and MTA, are represented by the peak of 3438.46 cm⁻^1^ in the HAp/MTA/nGO composite. GO is responsible for the C-H stretching vibrations at 2962.13 cm⁻^1^ and the C-O-C vibrations at 2096.24 cm⁻^1^. C=O extending from GO is probably represented by the peak at 1635.34 cm⁻^1^. The signal at 1384 cm⁻^1^ indicates the carbonate groups (CO₃^2^⁻) from MTA. The HAp exhibits distinctive phosphate (PO₄^3^⁻) vibrations at 1112.73 cm⁻^1^, 617.11 cm⁻^1^, and 464 cm⁻^1^. All three of the components (HAp, MTA, and nGO) are present in the composite, and their chemical interactions are demonstrated by the FTIR study. The effective synthesis of a HAp/MTA/nGO composite with a complex crystalline structure, porous shape, and chemical composition appropriate for possible bone-related applications is demonstrated by the combined XRD, SEM, and FTIR investigation. When these three elements are combined, they may have better mechanical, biological, and functional qualities than when they are used separately, which might improve the results of bone regeneration^47^.

There was a gradual increase in weight loss over time, with a total loss of **5.2 ± 1.1%** by day 28. indicates that the composite undergoes slow and controlled degradation, which is favorable for maintaining composite integrity during the initial stages of bone healing^48^.

The early phases of healing were indicated by the composite-treated defect’s first indications of bone in growth in the distal cortex two weeks after surgery. The control group, on the other hand, showed no discernible improvements, which is indicative of the critical-sized flaws’ inherent incapacity to mend on their own. By four weeks, the composite group showed excellent integration between the implant material and the bone stump, along with partial absorption of the implant material. Callus development surrounding the material surface suggested that the composite promoted tissue remodelling and cellular activity, indicating an active healing process. Conversely, the control group had very little change, and the defect stayed mostly the same^49^.

At eight weeks following surgery, the composite-treated group showed a considerable increase in newly formed bone that joined the ends of the bones and looked more radiopaque. This was the most notable change. Indicating that the composite encouraged both bone growth and structural integration, CT scans showed clear indications of remodelling, including the onset of canalisation. However, in line with earlier findings, the control group only showed a slight decrease in defect size and little new bone production in the distal cortex^23^.

According to previous research comparing several bone replacements, composites that contained bioactive substances like growth factors and hydroxyapatite have been found to increase osteogenic activity and speed up the healing process^50^**.** Furthermore, it has been shown that adding nanoparticles like nGO improved the mechanical characteristics and biological activity of composites, which improves overall bone regeneration results^51^. This implies that the HAp/MTA/nGO composite’s increased effectiveness in promoting bone repair may be attributed to the addition of nGO.

The histological analysis focused on the healing process, demonstrating that the composite treatment group had early indications of osteoblast development, cellular infiltration, and composite integration within the defect. In line with other observations, the newly created bone matrix was a significant improvement over the untreated critical-sized defect, which exhibited no meaningful bone regeneration, even if it was restricted and lacked the organized structure of normal bone^52^. This enhancement demonstrates how the composite may increase bone healing in non-healing lesions.

According to these results, the nGO/MTA/HAp composite has the potential as a bone replacement for deficiencies of a crucial size. In addition to the strong biocompatibility and integration with surrounding tissues, the composite is likely to offer an appropriate matrix for cell infiltration and the production of new bone, as the composite’s use in bone regeneration depends greatly on its capacity to promote tissue integration and cellular activity.

It is crucial to remember that the study was only carried out for a brief period up to 8 weeks, and that longer-term monitoring would be helpful to evaluate the whole healing and remodelling process. Further studies examining this composite’s efficacy in load-bearing applications and comparing it with other well-established bone graft materials would also yield important information about its potential in clinical settings. These investigations would aid in establishing the composite’s applicability for multiple clinical situations, as well as its effectiveness in fostering long-lasting bone repair.

## Conclusion

In conclusion, the nGO/MTA/HAp composite showed a greater potential as a bone substitute material for critical-sized lesions, along with improved bone growth and biocompatibility compared to untreated controls. These findings need further exploration and optimization of this composite for possible therapeutic uses in bone regeneration. To fully realize its promise in orthopedic surgery, further research is needed to focus on optimizing the composite’s composition and assessing its long-term stability.

## Data Availability

All data generated by this study are included in this manuscript. Any additional requests for information can be directed to, and will be fulfilled by, the corresponding authors.

## References

[1] Łuczak, J. W. et al. The future of bone repair: emerging technologies and biomaterials in bone regeneration. *Int. J. Mol. Sci.* **25**(23), 12766. 10.3390/ijms252312766 (2024). 39684476 10.3390/ijms252312766 PMC11641768

[2] Schmidt, A. H. Autologous bone graft: Is it still the gold standard?. *Injury* **52**, S18–S22. 10.1016/j.injury.2021.01.043 (2021). 33563416 10.1016/j.injury.2021.01.043

[3] Nashi, N. & Kagda, F. H. Current concepts of bone grafting in trauma surgery. *J. Clin. Orthop. Trauma.* **43**, 102231. 10.1016/j.jcot.2023.102231 (2023). 37636005 10.1016/j.jcot.2023.102231 PMC10448478

[4] Bezstarosti, H. et al. Insights into treatment and outcome of fracture-related infection: a systematic literature review. *Arch. Orthop. Trauma Surg.* **139**, 61–72. 10.1007/s00402-018-3048-0 (2019). 30343322 10.1007/s00402-018-3048-0 PMC6342870

[5] Flierl, M.A.et al. Outcomes and complication rates of different bone grafting modalities in long bone fracture nonunions: a retrospective cohort study in 182 patients. *J. Orthop. Surg. Res*. **8**, pp.1-10. 10.1186/1749-799X-8-33 )2013(. 10.1186/1749-799X-8-33 PMC3847297 24016227

[6] Eastlund, T. Viral infections are transmitted through tissue transplantation. In *Sterilisation of tissues using ionising radiations* (pp. 255-278). Woodhead Publishing. 10.1533/9781845690779.4.255 )2005(.

[7] Sohn, H.S. & Oh, J.K. Review of bone graft and bone substitutes with an emphasis on fracture surgeries. *Biomater. Res*. **23**(1), p.9. 10.1186/s40824-019-0157-y )2019(. 10.1186/s40824-019-0157-y PMC6417250 30915231

[8] Wiesmann, N. et al. Zinc oxide nanoparticles exhibit favorable properties to promote tissue integration of biomaterials. *Biomedicines*. **9**(10), p.1462. 10.3390/biomedicines9101462 )2021(. 10.3390/biomedicines9101462 PMC8533365 34680579

[9] Farjaminejad, S., Farjaminejad, R. & Garcia-Godoy, F. Nanoparticles in bone regeneration: a narrative review of current advances and future directions in tissue engineering. *J. Funct. Biomater.* **15**(9), 241. 10.3390/jfb15090241 (2024). 39330217 10.3390/jfb15090241 PMC11432802

[10] Cheng, J. et al. Graphene and its derivatives for bone tissue engineering: In vitro and in vivo evaluation of graphene-based scaffolds, membranes and coatings. *Front. Bioeng. Biotechnol.* **9**, 734688. 10.3389/fbioe.2021.734688 (2021). 34660555 10.3389/fbioe.2021.734688 PMC8511325

[11] Kazimierczak, P. & Przekora, A. Osteoconductive and osteoinductive surface modifications of biomaterials for bone regeneration: A concise review. *Coatings* **10**(10), 971. 10.3390/coatings10100971 (2020).

[12] Habibah, T.U., Amlani, D.V. & Brizuela, M. Hydroxyapatite dental material. https://www.ncbi.nlm.nih.gov/books/NBK513314/ (2018). 30020686

[13] Houschyar, K.S. et al. Wnt pathway in bone repair and regeneration–what do we know so far. *Front. Cell Dev. Biol*. **6**, p.170.10.3389/fcell.2018.00170 (2019). 10.3389/fcell.2018.00170 PMC6330281 30666305

[14] Lademann, F., Hofbauer, L.C. & Rauner, M. The bone morphogenetic protein pathway: the osteoclastic perspective. *​Front. Cell Dev. Biol*. **8**, p.586031. 10.3389/fcell.2020.586031 (2020). 10.3389/fcell.2020.586031 PMC7597383 33178699

[15] Grosso, A.et al. VEGF dose controls the coupling of angiogenesis and osteogenesis in engineered bone. *npj Regen. Med*. **8**(1), p.15. 10.1038/s41536-023-00288-1 (2023). 10.1038/s41536-023-00288-1 PMC10011536 36914692

[16] Zanotti, S. & Canalis, E. Notch signaling and the skeleton. *Endocr. Rev.* **37**(3), 223–253. 10.1210/er.2016-1002 (2016). 27074349 10.1210/er.2016-1002 PMC4890266

[17] Wei, H., Cui, J., Lin, K., Xie, J. & Wang, X. Recent advances in smart stimuli-responsive biomaterials for bone therapeutics and regeneration. *​Bone Res*. **10**(1), p.17. 10.1038/s41413-021-00180-y (2022). 10.1038/s41413-021-00180-y PMC8866424 35197462

[18] Schulze, F., Lang, A., Schoon, J., Wassilew, G. I. & Reichert, J. Scaffold guided bone regeneration for the treatment of large segmental defects in long bones. *Biomedicines.* **11**(2), 325. 10.3390/biomedicines11020325 (2023). 36830862 10.3390/biomedicines11020325 PMC9953456

[19] Jeong, J., Kim, J. H., Shim, J. H., Hwang, N. S. & Heo, C. Y. Bioactive calcium phosphate materials and applications in bone regeneration. *Biomaterials research.* **23**(1), 4. 10.1186/s40824-018-0149-3 (2019). 30675377 10.1186/s40824-018-0149-3 PMC6332599

[20] Eskandarinezhad, M. Effect of incorporating hydroxyapatite and zinc oxide nanoparticles on the compressive strength of white mineral trioxide aggregate. *​J. Dent*. **21**(4), p.300. 10.30476/DENTJODS.2020.82963.1034 (2020). 10.30476/DENTJODS.2020.82963.1034 PMC7737922 33344680

[21] Zaaba, N. I. et al. Synthesis of graphene oxide using modified hummers method: solvent influence. *Procedia Eng.* **184**, 469–477. 10.1016/j.proeng.2017.04.118 (2017).

[22] Hanifah, M.F.R. et al. Synthesis of graphene oxide nanosheets via modified Hummersâ€™ method and its physicochemical properties. *​J. Teknol. (Sci. Eng.)​*.**74**(1). 10.11113/jt.v74.3555 (2015).

[23] Jiříčková, A., Jankovský, O., Sofer, Z. & Sedmidubský, D. Synthesis and applications of graphene oxide. *Mater.* **15**(3), p.920.10.3390/ma15030920 (2022). 10.3390/ma15030920 PMC8839209 35160865

[24] Liu, J., Zhou, P., Long, Y., Huang, C. & Chen, D. Repair of bone defects in rat radii with a composite of allogeneic adipose-derived stem cells and heterogeneous deproteinized bone. ​Stem Cell Res. Ther. **9**, pp.1-10. 10.1186/s13287-018-0817-1 (2018). 10.1186/s13287-018-0817-1 PMC5870513 29587852

[25] Cunningham, B. P., Brazina, S., Morshed, S. & Miclau, T. III. Fracture healing: A review of clinical, imaging and laboratory diagnostic options. *Injury* **48**, S69–S75. 10.1016/j.injury.2017.04.020 (2017). 28483359 10.1016/j.injury.2017.04.020

[26] Su, D.et al. Dual-energy computed tomography and micro-computed tomography for assessing bone regeneration in a rabbit tibia model. ​Sci. Rep. **14**(1), p.5967. 10.1038/s41598-024-56199-8 (2024(. 10.1038/s41598-024-56199-8 PMC10933353 38472263

[27] Di Carlo, S. et al. Histological and immunohistochemical evaluation of mandibular bone tissue regeneration. nt. J. Immunopathol. Pharmacol. **32**, p.2058738418798249. 10.1177/2058738418798249 (2018). 10.1177/2058738418798249 PMC6201177 30350738

[28] Gibson-Corley, K. N., Olivier, A. K. & Meyerholz, D. K. Principles for valid histopathologic scoring in research. *Vet. Pathol.* **50**(6), 1007–1015. 10.1177/0300985813485099 (2013). 23558974 10.1177/0300985813485099 PMC3795863

[29] Mohammed, F.M., LM, A., Shareef, A.M., & MG, T. Evaluation the effect of high and low viscosity Nano-hydroxylapatite gel in repairing of an induced critical-size tibial bone defect in dogs: Radiolographical study. *​J. Appl. Vet. Sci*. **8**(3), pp.105-110. 10.21608/javs.2023.215990.1239 (2023).

[30] Sagi, H.C. & Patzakis, M.J. Evolution in the acute management of open fracture treatment? Part 1. ​J. Orthop. Trauma. **35**(9), pp.449-456. 10.1097/BOT.0000000000002094 (2021). 10.1097/BOT.0000000000002094 34415869

[31] Komaki, H., Tanaka, T., Chazono, M. & Kikuchi, T. Repair of segmental bone defects in rabbit tibiae using a complex of β-tricalcium phosphate, type I collagen, and fibroblast growth factor-2. *Biomaterials* **27**(29), 5118–5126. 10.1016/j.biomaterials.2006.05.031 (2006). 16769112 10.1016/j.biomaterials.2006.05.031

[32] Xie, C. et al. Recombinant human bone morphogenetic protein is a valid alternative to autologous bone graft for long bone non-unions: a systematic review and meta-analysis. *Surgeon.* **21**(4), e173–e182. 10.1016/j.surge.2022.11.004 (2023). 36682906 10.1016/j.surge.2022.11.004

[33] Moon, Y.J., Jeong, S. & Lee, K.B. Bone morphogenetic protein 2 promotes bone formation in bone defects in which bone remodeling is suppressed by long-term and high-dose zoledronic acid. *Bioengineering*. **10**(1), p.86. 10.3390/bioengineering10010086 )2023(. 10.3390/bioengineering10010086 PMC9854702 36671658

[34] Kungvarnchaikul, I., Subbalekha, K., Sindhavajiva, P.R. & Suwanwela, J. Deproteinized bovine bone and freeze‐dried bone allograft in sinus floor augmentation: A randomized controlled trial. ​Clin. Implant Dent. Relat. Res. **25**(2), pp.343-351. 10.1111/cid.13179 )2023(. 10.1111/cid.13179 36628938

[35] Pountos, I., Georgouli, T., Bird, H., Kontakis, G. & Giannoudis, P.V. The effect of antibiotics on bone healing: current evidence. *Expert Opin. Drug Saf*. **10**(6), pp.935-945. 10.1517/14740338.2011.589833) 2011(. 10.1517/14740338.2011.589833 21824037

[36] Pires, J.L.D.S. Repair of critical size bone defects using synthetic hydroxyapatite or xenograft with or without the bone marrow mononuclear fraction: A histomorphometric and immunohistochemical study in rat calvaria. Materials. **14**(11), p.2854. 10.3390/ma14112854 )2021(. 10.3390/ma14112854 PMC8199028 34073482

[37] Tay, F.R. Bioactivity of mineral trioxide aggregate and mechanism of action. Mineral trioxide aggregate in dentistry: from preparation to application. pp.61-85. 10.1007/978-3-642-55157-4_4) 2014(.

[38] Xing, J. & Liu, S. Application of loaded graphene oxide biomaterials in the repair and treatment of bone defects: a review. ​Bone Jt. Res. **13**(12), p.725. 10.1302/2046-3758.1312.BJR-2024-0048.R1 (2024(. 10.1302/2046-3758.1312.BJR-2024-0048.R1 PMC11617066 39631429

[39] Kashte, S.B.et al. Osteoinductive potential of graphene and graphene oxide for bone tissue engineering: a comparative study. *​J. Orthop. Surg. Res*. **19**(1), p.527.10.1186/s13018-024-05028-9 (2024). 10.1186/s13018-024-05028-9 PMC11365281 39215309

[40] Ozder, M. N., Çiftçi, F., Rencuzogullari, O., Arisan, E. D. & Ustündag, C. B. In situ synthesis and cell line studies of nano-hydroxyapatite/graphene oxide composite materials for bone support applications. *Ceram. Int.* **49**(9), 14791–14803. 10.1016/j.ceramint.2023.01.075 (2023).

[41] Zhou, H. et al. Exploring the application of Graphene Oxide-based nanomaterials in the repair of osteoporotic fractures. *Nanomaterials* **14**(6), 553. 10.3390/nano14060553 (2024). 38535701 10.3390/nano14060553 PMC10976089

[42] Tawil, P.Z., Duggan, D.J. & Galicia, J.C. MTA: a clinical review. *​Compend. Contin. Educ. Dent (Jamesburg, NJ: 1995)*. **36**(4), p.247. https://pubmed.ncbi.nlm.nih.gov/25821936/ (2015). PMC4962539 25821936

[43] Jin, Y. et al. Oxidative stress-induced apoptosis of osteoblastic MC3T3-E1 cells by hydroxyapatite nanoparticles through lysosomal and mitochondrial pathways. *RSC Adv.* **7**(21), 13010–13018. 10.1039/C7RA01008G (2017).

[44] Muthu, M.S., Xavier, S.S.J., Ajith, P. & Anand, D.P. Preparation and characterization studies of nano graphene oxide. *Mater. Today: Proc*. **66**, pp.2449-2454. 10.1016/j.matpr.2022.06.367 )2022(.

[45] Akhavan, H., Mohebbi, P., Firouzi, A. & Noroozi, M. X-Ray diffraction analysis of ProRoot mineral trioxide aggregate hydrated at different pH values. *Iran. Endod. J*. **11**(2), p.111. 10.7508/iej.2016.02.007 )2016(. 10.7508/iej.2016.02.007 PMC4841345 27141218

[46] Barros, C.M.B., de Oliveira, S.V., Marques, J.B., de Souto Viana, K.M. & de Melo Costa, A.C.F. November. Analysis of the hydroxyapatite incorporate MTA dental application. Mater. Sci. Forum*.* **727**, pp. 1381-1386. Trans Tech Publications Ltd. 10.4028/www.scientific.net/MSF.727-728.1381 (2012).

[47] Arpacay, B.M. et al. Resveratrol-loaded PCL-PEG/GO/HAP biocomposite bone membranes: Evaluation of mechanical properties, release kinetics, and cellular response. *​J. Appl. Biomater. Funct. Mater*. **23**, p.22808000251314087. 10.1177/22808000251314087 (2025). 10.1177/22808000251314087 39894962

[48] Mohamed, K.R., Beherei, H.H. & El-Rashidy, Z.M. In vitro study of nano-hydroxyapatite/chitosan–gelatin composites for bio-applications. *​J. Adv. Res*. **5**(2), pp.201-208. 10.1016/j.jare.2013.02.004 (2014). 10.1016/j.jare.2013.02.004 PMC4294712 25685488

[49] Gadallah, S.M., Abd-Elkawi, M., Misk, T.N. & Sharshar, A.M. The efficacy of nano-calcium carbonate derived from coral reefs and nano-silver to induce new bone formation in critical radial bone defect in rabbits: Radiological evaluation. *Journal of Current Veterinary Research*. **4**(2), pp.113-123. 10.21608/jcvr.2022.267519 (2022).

[50] van Houdt, C. I. et al. Regenerating critical size rat segmental bone defects with a self-healing hybrid nanocomposite hydrogel: effect of bone condition and BMP-2 incorporation. *Macromol. Biosci.* **21**(8), 2100088. 10.1002/mabi.202100088 (2021). 10.1002/mabi.202100088 34117838

[51] Ciobanu, P. et al. Experimental Study on Rats with Critical-Size Bone Defects Comparing Effects of Autologous Bone Graft, Equine Bone Substitute Bio-Gen® Alone or in Association with Platelet-Rich Fibrin (PRF). *Polymers* **16**(11), 1502. 10.3390/polym16111502 (2024). 38891449 10.3390/polym16111502 PMC11175103

[52] Abd-Elkawi, M., Sharshar, A., Misk, T., Elgohary, I. & Gadallah, S. Effect of calcium carbonate nanoparticles, silver nanoparticles and advanced platelet-rich fibrin for enhancing bone healing in a rabbit model. *Sci. Rep* **13**(1), 15232. 10.1038/s41598-023-42292-x (2023). 37709814 10.1038/s41598-023-42292-x PMC10502137

